# Physically unclonable functions taggant for universal steganographic prints

**DOI:** 10.1038/s41598-022-04901-z

**Published:** 2022-01-19

**Authors:** Takao Fukuoka, Yasushige Mori, Toshiya Yasunaga, Kyoko Namura, Motofumi Suzuki, Akinobu Yamaguchi

**Affiliations:** 1 Department of Micro Engineering, Kyoto University, Kyoto Daigaku-Katsura, Nishikyo-ku, Kyoto, 615-8540 Japan; 2 Department of Chemical Engineering and Materials Science, Doshisha University, 1-3 Tatara Miyakodani, Kyotanabe-shi, Kyoto, 610-0394 Japan; 3 Laboratory of Pharmaceutical Engineering, School of Pharmacy, Aichi Gakuin University, 1-100 Kusumoto-cho, Chikusa-ku, Nagoya, Aichi 464-8650 Japan; 4 Laboratory of Advanced Science and Technology, University of Hyogo, 3-1-2 Kouto, Kamigori, Ako-gun, Hyogo, 678-1205 Japan

**Keywords:** Engineering, Materials science, Nanoscience and technology, Optics and photonics

## Abstract

Counterfeiting of financial cards and marketable securities is a major social problem globally. Electronic identification and image recognition are common anti-counterfeiting techniques, yet they can be overcome by understanding the corresponding algorithms and analysis methods. The present work describes a physically unclonable functions taggant, in an aqueous-soluble ink, based on surface-enhanced Raman scattering of discrete self-assemblies of Au nanoparticles. Using this stealth nanobeacon, we detected a fingerprint-type Raman spectroscopy signal that we clearly identified even on a business card with a pigment mask such as copper-phthalocyanine printed on it. Accordingly, we have overcome the reverse engineering problem that is otherwise inherent to analogous anti-counterfeiting techniques. One can readily tailor the ink to various information needs and application requirements. Our stealth nanobeacon printing will be particularly useful for steganography and provide a sensitive fingerprint for anti-counterfeiting.

## Introduction

Counterfeit goods account for 3.3% of world trade value and financial losses of USD 509 billion (EUR 460 billion), based on data for 2016^1–3^. Accordingly, counterfeit-enabling technology is so valuable that reverse engineering remains an ongoing challenge. Conventional anti-counterfeit technology has two problems: one must imitate the authentication system, and doing so can be time-consuming and costly when the system is complex. Consequently, there is a need for anti-counterfeiting technology that overcomes these problems.

Digital authentication systems in integrated-circuit-equipped credit cards and electronic commerce with blockchain technology via the internet^4^ are compatible with robust security systems that are used all over the world. However, because 20.4 billion Internet of Things devices were connected via the internet in 2020^5^, the cyber world is closely integrated with the physical world. In modern society—including human activities such as transport; commerce; and use of goods, food, and drugs—the risk of cyber-attacks is considerable. Therefore, in addition to cyber security, physical identification is an anti-counterfeit technology for goods that are distributed, traded, and used. Recently, proposals have also been demonstrated to prevent counterfeiting by photodetection using the nanostructured metasurfaces^6,7^.

For secure identification, physically unclonable functions (PUFs) with a specific challenge-response that is dependent on the fingerprint properties of the device are indispensable^4,8–11^. PUFs combined with chemical methods that are compatible with well-established nanotechnology are an active area of research^1,12–14^. PUFs have been intrinsically limited to electronic and mechanical devices, but currently PUFs are also being applied as various taggants to soft materials, such as printed devices^13^ and organic semiconductors^14^. For anti-counterfeit measures, these PUF taggants are identified by analytical methods based on the properties of the taggants instead of specific challenge-responses of the devices.

PUF taggants are compatible with numerous analytical detection procedures. Because extremely sensitive analysis on a small quantity of PUFs is desirable for quick identification and minimal cost, surface-enhanced Raman scattering (SERS) detection is promising for PUF taggants. For example, Mirkin’s group demonstrated an encoding system based on dispersible arrays of nanodisks prepared by lithography and functionalized with SERS-active chromophores^15–17^. Their nanodisk arrays can be encoded both in terms of the physical arrangements of the chromophores and by SERS spectroscopy. Natan and coworkers proposed encryption based on silica-encapsulated noble metal nanoparticles with Raman-active molecules and SERS detection^18,19^. Ling’s group fabricated SERS-active nanowires and nanopillars by lithography and demonstrated anisotropic plasmonic image printing^20,21^. Tian et al. fabricated a SERS-active polymer film with silver-coated Au nanorods^22^.

Fukuoka et al. reported discrete self-assemblies of Au nanoparticles (AuNPs) and a reporter molecule; and demonstrated long-lived, on-dose authentication of commercial tablets^23^. In this manner, nanoparticles assembles are common in SERS PUFs^24–29^. Multiplex encoding is important for PUF taggants; SERS taggants have the advantage of enabling researchers to choose the reporter molecule from a diverse set of Raman-active molecules. For example, Gu’s group demonstrated a three-dimensional encoding capacity of greater than 3 × 10^15051^ by drop-casting SERS tags and a coarse-grained method^30^.

Arppe and Sørensen recently reviewed such SERS taggants^12^. In their review, they described “*Inks and engraved structures are modern and highly advanced extensions of historical anti-counterfeiting systems. The same is true for approaches that combine nanostructured surfaces containing chemical probes with surface-enhanced Raman scattering (SERS) signatures. However, all of these approaches rely on deterministic tags and can therefore be copied by counterfeiters.*” Even though SERS enables label-free and trace-level molecular detection^31–38^, it requires noble metal nanostructures, the presence of which is a vulnerability that counterfeiters can use as a starting point for reverse engineering PUF taggants.

To overcome the reverse engineering problem in a practical manner, we used a nanobeacon aqueous-soluble ink based on SERS. The nanobeacon comprises discrete, aperiodic structure that induced by a contingence. Our chain aggregates are primitive structures but firmly emit SERS. Due to its discretely distributed nanostructure, it is less likely to be found by malicious intent than regular and sophisticated structures made by lithography or encapsulation. If not found, it will not be reverse engineered. The nanobeacons can be dispersed in an aqueous solution to mask the presence of this PUF taggant in the printed material and prevent reverse engineering because reporter molecule randomly distributed and included in the chain like AuNPs aggregates. We have thus developed a stealth nanobeacon that facilitates steganographic embedded printing^39,40^. For SERS applications, we applied a stealth nanobeacon in an aqueous-soluble ink to anti-counterfeiting. Our technology does not depend on deterministic tags and exhibits stealth features that one can observe in the ink.

## Results and discussion

**Figure 1:**
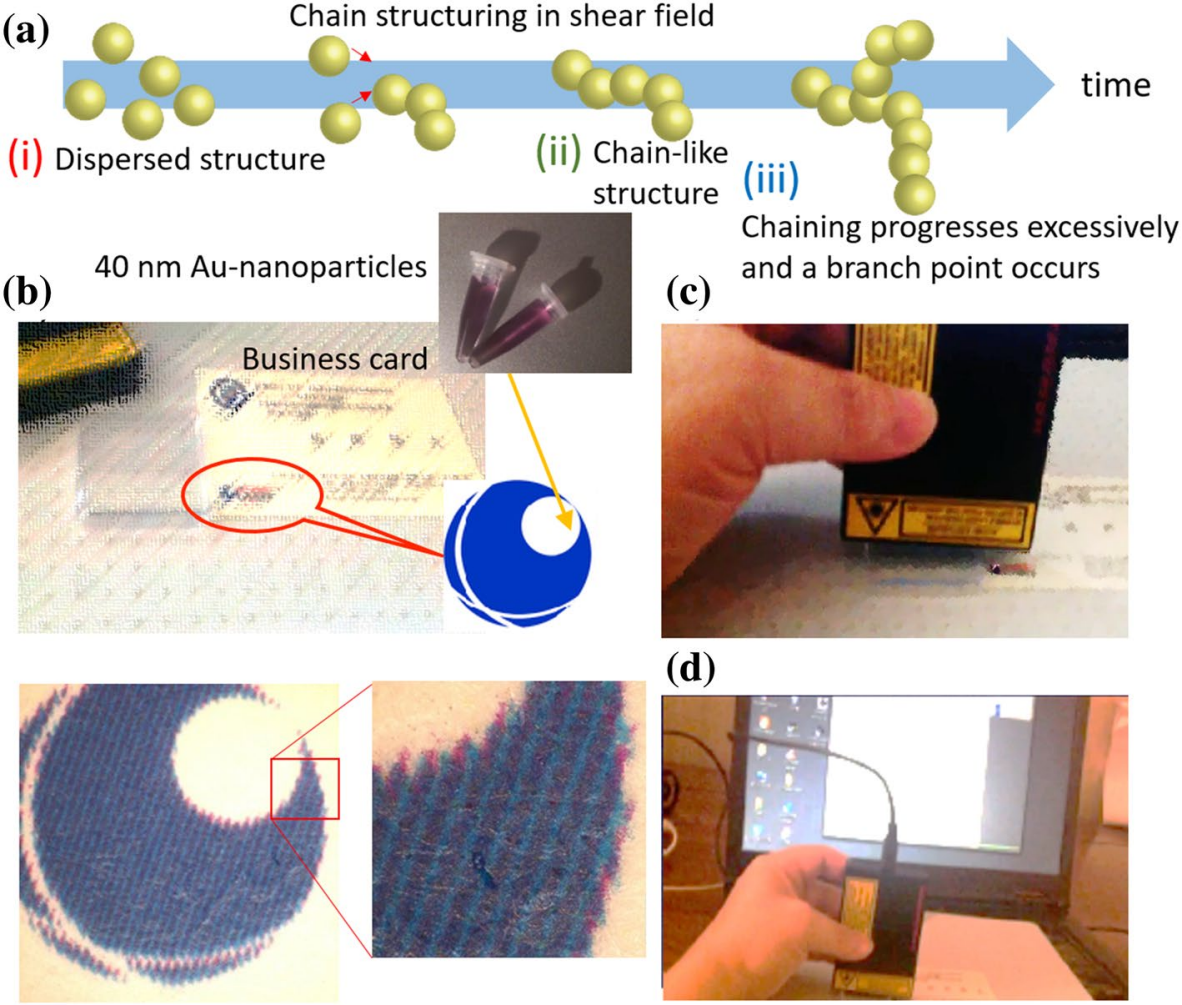
Experimental overview: (**a**) Schematic of the time evolution of Au nanoparticles aggregates and formation of a chain structure. With time evolution under the shear field, the following nanostructured aggregates are sequentially formed: (i) dispersed structure, (ii) chain-like structure, and (iii) aggregates structure with a branch point. (**b**) Magnified optical photograph of a business card with ink containing stealth nanobeacons, and the position of the spot. (**c**) Raman spectroscopic measurement of the spotting position and (**d**) spectrum detection result from the ink, including the stealth nanobeacons.

We fabricated the nanobeacons by bottom-up, diffusion-limited aggregation of colloidal AuNPs under a shear force of time-controlled agitation (Fig. 1a)^41–49^. Aqueous suspensions of AuNPs were synthesized using a standard citrate reduction protocol^50^. Accordingly, we mixed 4, 4ʹ-bipyridine (4bpy) molecules into the stealth nanobeacons. Because chain-like aggregates reported by Creighton’s group^48^ spontaneously form in the shear field^41–49^, the stealth nanobeacons exhibited the strong SERS signal of 4bpy. We spray-coated the nanobeacons and used a shadow mask to obtain the desired positions (Fig. 1b). We deposited the nanobeacons in a blue area, printed with a copper phthalocyanine pigment. Even magnifying the observation area with an optical microscope did not reveal the presence of the stealth nanobeacons. Next, we used a portable Raman spectroscope (C13650, Hamamatsu Photonics Corporation; laser excitation wavelength: 785 nm, laser spot size: $\sim$ 0.8 mm $\times$ 0.8 mm) connected to a smartphone or laptop personal computer to image the place where we applied the stealth nanobeacons (Fig. 1c). We readily and quickly observed the Raman signal from 4bpy (Fig. 1d).

**Figure 2:**
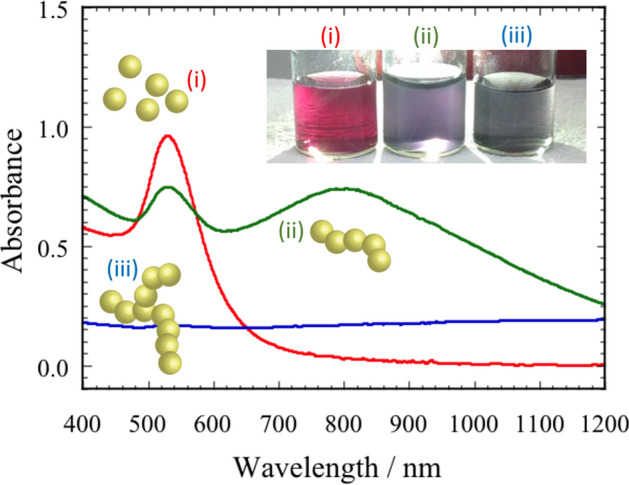
Typical absorption spectra of Au nanoparticles: (i) dispersed structure, (ii) chain-like structure, and (iii) precipitated aggregates structure with a branch point.

We next discuss the optical properties of the stealth nanobeacons. Figure 2 shows the structure-dependence of the optical absorbance for three typical structures: (1) AuNPs dispersed without agglomeration; (2) stealth nanobeacons, which we meta-stabilized by diffusion-limited aggregation; and (3) unstable and precipitated aggregates. Each schematic denotes the corresponding typical nanostructure in Fig. 2. These structures are made separately by controlling the time when the shear field is applied^41–49^. Evaluation of the absorption spectrum enables us to distinguish the cases (1)–(3). Regarding case (1), AuNPs dispersed without agglomeration, we observed a single resonance (red solid line). This peak resonance wavelength was at ~ 540 nm. Regarding case (2), stealth nanobeacons, we observed the other red-shifted resonance at ~ 800 nm. We attribute this red-shifted resonance to coupled localized surface plasmon resonance, which can generate SERS with a two-fold electromagnetic enhancement^41,45–47,51,52^. According to Le Ru and Etchegoin^38^, the red-shifted resonance arises from dipolar interactions or dipolar coupling between the two single-sphere dipolar localized surface plasmon resonances. The red-shift depends on the strength of the interactions and therefore increases in accordance with decreasing gap sizes between the AuNPs. Taylor et al. also reported that if nanoparticles with a diameter of 30 nm and excitation wavelength of 685 nm, the SERS intensity is maximal around 3 or 4 particles, corresponding to the length for which the chain resonance is close to the illumination wavelength. For illumination in the near-infrared, 785 nm, the detuning diminishes with increasing particle number, and the enhancement is optimized for the larger chains. However, as shown in Fig. 2 and other reports^41,45–47^, if the chain has multiple branch points and is networked, the SERS intensity drastically decreases. In the stealth nanobeacons, we formed chain-like nanostructures [(ii) of Fig. 2] and confined 4bpy between the gaps in the assemblies, as we have reported in previous work^45–47,49,51^. Broad resonances are attributable to higher-order interactions from the chain-like nanostructures comprising AuNPs. Regarding case (iii) of Fig. 2, precipitating aggregates (blue solid line), the absorbance was independent of the wavelength and we observed no resonance. This result arises because the aggregates became too large to remain in a dispersed state; the localized surface plasmon resonance became undetectable in this measurement range^45–47^.

**Figure 3:**
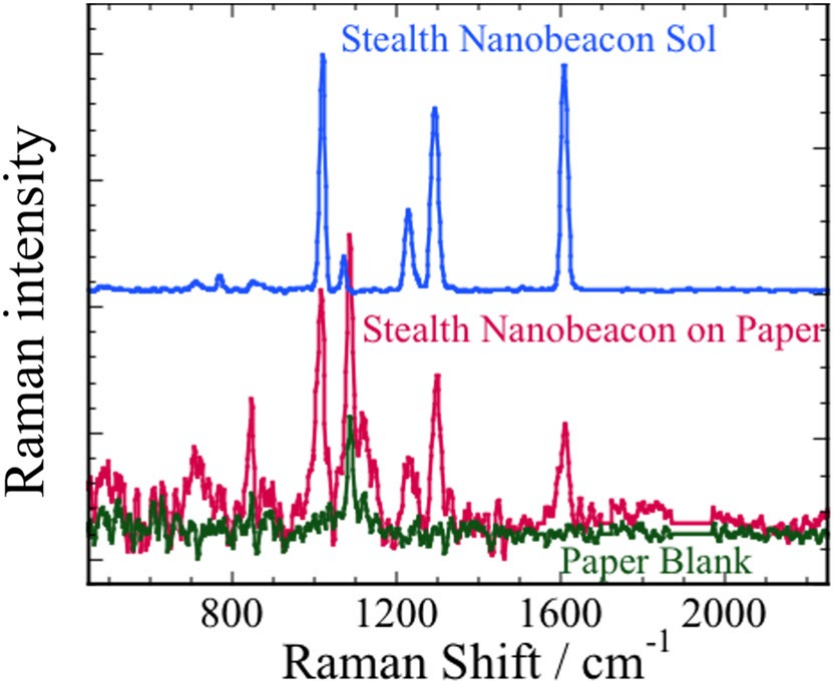
Surface-enhanced Raman spectra of stealth nanobeacon sol (blue), stealth nanobeacons on paper (red), and paper blank (green).

Figure 3 shows SERS spectra for the following: (1) stealth nanobeacon sol (blue solid line), (2) stealth nanobeacons on paper (red solid line), and (3) blank paper (green solid line). The SERS peak positions in condition 2 agree with those in condition 1, whereas we mainly observed the Raman peak of calcium carbonate^53^ loading filler in condition 3 (Fig. 3). These results indicate that the stealth nanobeacons can help prevent the forgery of printed materials such as financial cards and marketable securities.

Other SERS or Raman signals might also depend on the superposition of the dyes, pigments, and fillers from blank paper (Fig. 3). In particular, copper phthalocyanine, known as phthalocyanine blue, exhibits a strong absorption in the near-infrared and strong Raman peaks^39,40^. Phthalocyanine blue is a common blue color in printed materials because of properties such as light fastness, tinting strength, covering power, and resistance to alkalis and acids. In the following demonstration, to obtain a deeper understanding of the properties of the stealth nanobeacons, we performed more-detailed SERS spectrum analyses and comparative studies. We deposited the stealth nanobeacons over a blue area on a business card, painted with copper phthalocyanine pigment (Fig. 1).

**Figure 4:**
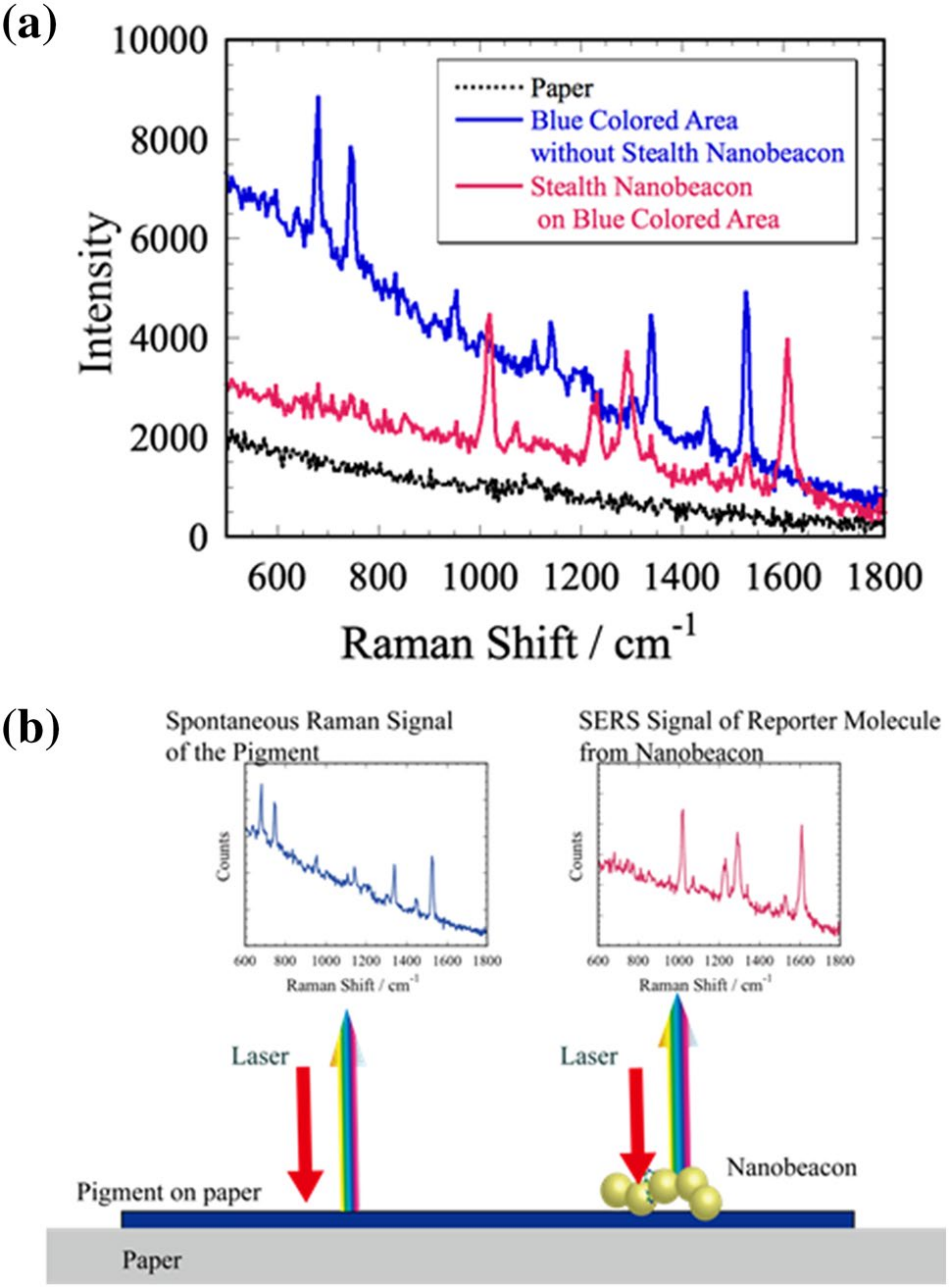
Surface-enhanced Raman spectroscopy measurements on three positions, and the corresponding measurement scheme: (**a**) Spectra of stealth nanobeacons on the blue area of a name card (red solid line), blue area of the name card without the nanobeacons (blue solid line), and paper (black dotted line). (**b**) Scheme of the measurements indicating.

We obtained SERS spectrum measurements on three positions: where nothing was printed on the business card, the blue area, and the place where we deposited the nanobeaon over the blue area. Accordingly, we obtained Raman spectra (Fig. 4a) of the nonprinted area and the blue area without the nanobeacon (black dotted and blue solid lines, respectively). No conspicuous Raman resonance structure was evident in the nonprinted area without the nanobeacon. We clearly observed a Raman spectrum from the blue area without the nanobeacon; the Raman peak positions at 686, 752, 959, 1013, 1148, 1344, 1451, and 1527 cm^−1^ are consistent with phthalocyanine blue (PB15) K-23050^54^. In contrast, we observed the characteristic enhanced Raman spectrum (red solid line) of 4bpy (1000, 1200, 1280, and 1600 cm^−1^) from the stealth nanobeacons deposited on the blue area of the business card and detected slight Raman signals from phthalocyanine blue (Fig. 4a). The reason for this is considered to be the relationship between the laser spot size of the Raman spectroscope and the size of the spotted area of nanobeacon ink. The laser spot size is less than about 0.8 mm $\times$ 0.8 mm, while the size of the spotted area is about 0.5 mm $\times$ 0.5 mm or less. That is, signals from the areas other than the area where the nanobeacon ink is dotted are superimposed. Even a small amount of nanobeacon will generate a stronger signal than the background signal. This fact is considered to be a very useful in terms of application for the rapid-easy authenticity judgment authentication. In this demonstration, the mobile Raman spectroscope was used, and Raman spectroscopy was performed by hand. The focal length and measurement angle changed depending on the measurement location and camera shake. Consequently, the background intensity of each line is different. Even if the background intensity changed as shown in Fig. 4a, the signal from the nanobeacon ink can be noticeably expressed, indicating that it will provide a very easy-to-use operation.

We clearly distinguished our stealth nanobeacon, overlaid on the printed pigment, from the pigment components printed on the printed materials. SERS is common in pigment analyses of printed materials^55^; but in this study, by depositing the stealth nanobeacon over the printed pigment, the pigment was not evident by Raman spectroscopy. Concomitantly, the color of the pigment reduced the visibility of the overlaying stealth nanobeacon. One can use the stealth nanobeacons as a hidden ink without interference from the pigment in the printed area (Fig. 4b).

**Figure 5:**
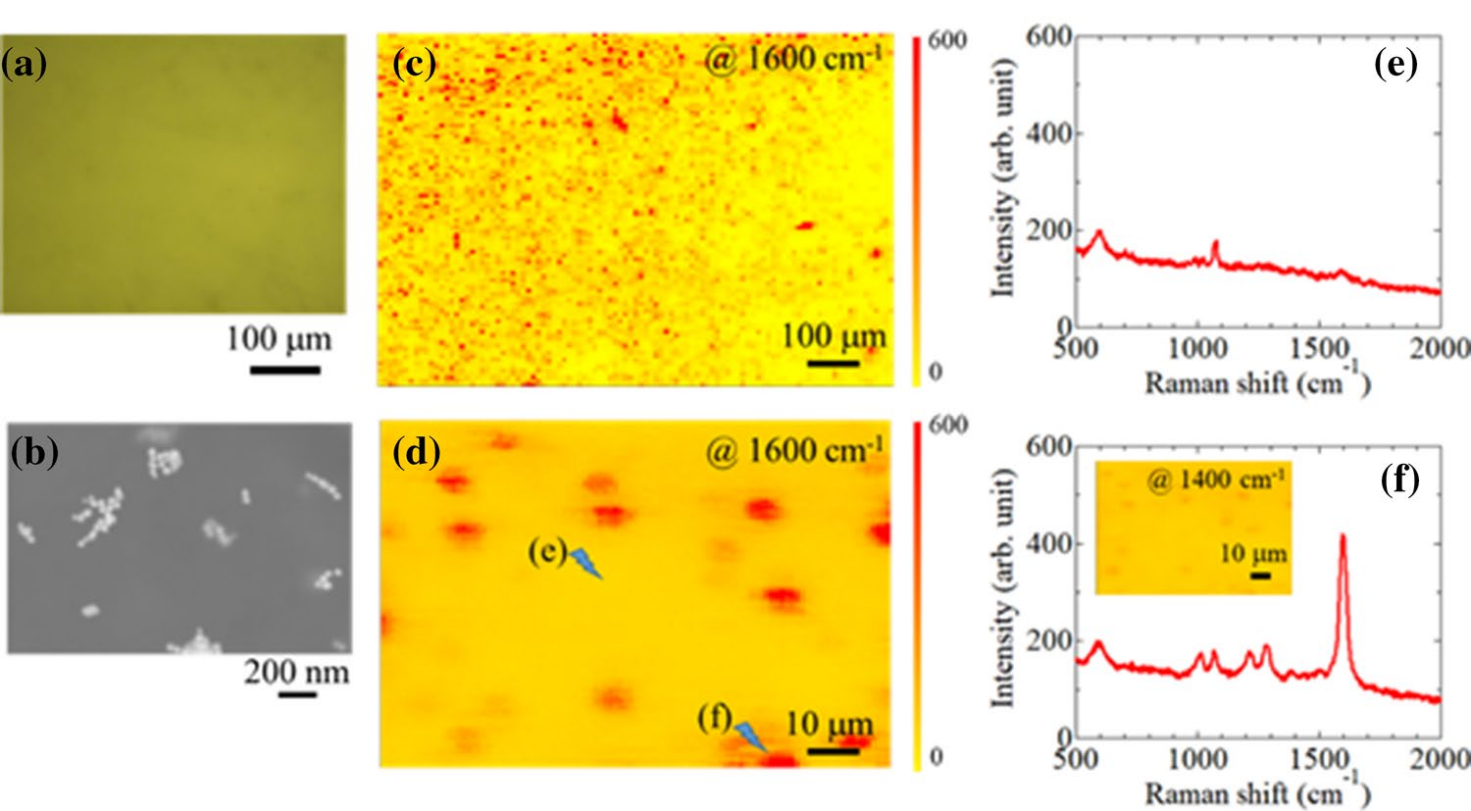
Distribution of the nanobeacons: (**a**) Optical photograph of the paper surface to which we applied aqueous ink that included nanobeacons. (**b**) Scanning electron microscopy image of Au nanoparticle aggregates included in the stealth nanobeacon. Surface-enhanced Raman spectroscopy (SERS) mappings of (**c**) 1 mm × 1 mm and (**d**) 100 μm × 100 μm at 1600 cm^−1^. (**e**,**f**) SERS spectra for the locations in the Raman mapping shown in (**d**). The inset in (**f**) shows a SERS mapping at (**d**) at 1400 cm^−1^.

To further investigate the characteristics of this nanobeacon ink, we evaluated the distribution of the nanobeacons—after applying them to coated paper—by, for example, microscopic Raman spectroscopy, optical microscope observations, and Raman mapping. Figure 5a shows an optical micrograph of the paper surface. The surface appears to be smooth and uniform. Figure 5b shows high-resolution scanning electron microscope observations. The particles were in a discrete chain-like morphology. This result agrees with the conceptual diagram of Fig. 1a. Figure 5c,d show Raman mapping images in 1 mm × 1 mm and 100 μm × 100 μm regions, respectively, at a Raman shift of 1600 cm^−1^. Figure 5e,f show Raman spectra at positions e and f in Fig. 5d, respectively. The regions of high SERS intensity were lumps of ~ 8–15 µm and were randomly distributed (Fig. 5d). The following three factors contributed to the SERS intensities being relatively well-spread as several large lumps: (1) the relatively larger agglomerates consisting of several nanobeacons were mixed in the ink and non-uniformly spatial distribution of the agglomerates occurred, (2) the ink soaked into the porous paper and diffused to form lumps, and (3) the laser spot of the Raman microscope that we used for this measurement was several micrometres in diameter. Other contributing factors are possible and are a focus of ongoing research. By printing the nanobeacon ink in this manner, we embedded information in only the narrow area to which we applied the ink. The embedded information was randomly distributed; an ink that emits a strong SERS signal is useful as a versatile anti-counterfeiting technology.

**Figure 6:**
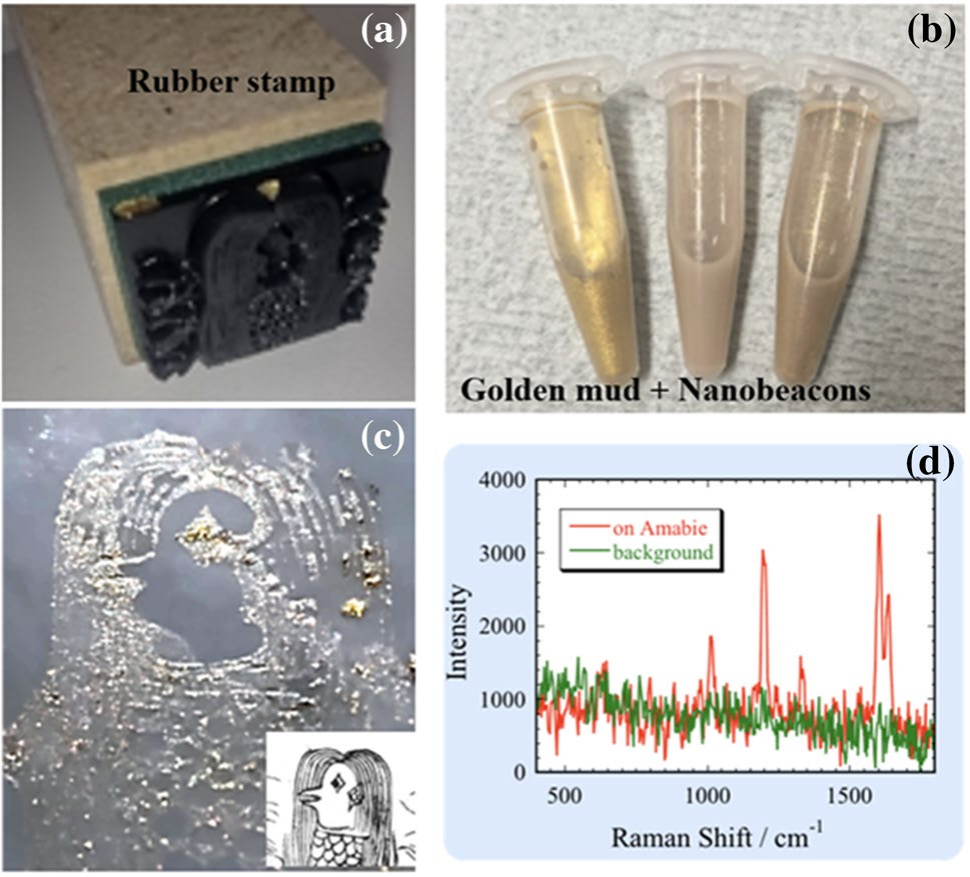
Example of hidden embedded information: (**a**) Rubber stamp with Amabie engraved. (**b**) Stamp ink comprising Au mud paint and nanobeacons. (**c**) Stamped Amabie. Inset: original figure. (**d**) Raman spectra of Amabie and background. We processed the Amabie portrait from a photograph courtesy of the Main Library of Kyoto University.

Finally, we demonstrated an example of hidden embedded information that we fused with Japanese traditional crafts. By mixing this nanobeacon ink with Au mud paint used for drawing Au folding screens, we embedded—in an invisible manner—the Raman spectrum in the picture drawn with Au paint. In this nanobeacon ink, we embedded 4, 4’-Vinylenedipyridine (BPE). The picture shown here is the Japanese supernatural spirit Amabie^56^. Here, we demonstrated the Amabie stamps can include plasmonic information. Figure 6a shows a rubber stamp that depicts Amabie. Figure 6b shows an ink mixed with Au mud paint. Figure 6c shows this ink stamped onto paper (the inset shows the original drawing). Au mud paint is usually stamped in this manner. Although the nanobeacon is evident with unaided eyes, the Raman spectral information is embedded and not evident (Fig. 6c). Figure 6d shows the corresponding Raman spectrum. In this manner, by using the nanobeacon ink, one can print a figure. In fact, the structure reminiscent of expressing SERS cannot be observed in neither the 50 $\times$ objective lens image of Fig. 5a nor 10 $\times$ objective lens images of SFigs. 1 and 2 in the supplementary information. As shown in the SFigs. 1–3, it is clear that SERS appears from only the paste. Repeatedly, our nanobeacon is so small that it is randomly dispersed in space, so that it cannot be seen with the naked eye, and even if it is observed by SEM or Transmission Electron Microscopy (TEM), it has an aperiodic chain structure and cannot be reverse engineered. By combining multiple types of ink, extremely complex cryptographic information can be included in the figure.

Our nanobeacon ink facilitates incorporation of Raman signals that one can embed in various compositions, not limited to paints and drawings. Combining Raman spectroscopy with the stealth nanobeacons will facilitate development of a versatile steganography print.

## Conclusions

We applied invisible stealth nanobeacons to steganographic prints. One can use this stealth nanobeacon as an aqueous-soluble ink. The SERS signals are strong enough that a fingerprint-like signal is clearly distinguishable without interference from the pigments in the printed area. One can readily tailor the stealth nanobeacons with 4bpy or other molecules as desired. Therefore, one can readily design a stealth nanobeacon in accordance with the application and desired embedded information.

The potential of this development to anti-counterfeiting is especially noteworthy. A true–false test system in combination with a Raman spectrometer can readily authenticate the stealth nanobeacons. Therefore, this development is a unique technology for developing security features that rely on steganography, and can provide fingerprint-type watermarking for anti-counterfeiting. The quantity of the AuNPs used in the stealth nanobeacons was small: on the order of nanograms. There is no need for time-consuming and expensive syntheses for bottom-up self-assembly. Therefore, we have facilitated development of a next-generation anti-counterfeiting system.

## Methods summary

Aqueous suspensions of AuNPs were prepared according to the standard citrate reduction protocol^50^. Shearing conditions were optimized to control cluster chain length and distribution as described by refs. 41 and 49. After purification, the separated particles were dispersed in ultrapurewater containing 50 nM 4, 4’-bipyridine (4bpy) molecules and bound 4bpy with AuNPs to convert the functionalities on the AuNPs chains, resulting in creating the stealth nanobeacons. This suspension was completely created in all self-assembly process and was spayed through a shadow mask to the desired positions or areas. The Raman spectra were obtained using a simple Raman spectrometer with 785 nm excitation laser.

## Supplementary Information

Supplementary Information.
