# Supplementary material for: Physically unclonable functions taggant for universal steganographic prints
Source: Sci Rep. 2022 Jan 19;12:985. doi: 10.1038/s41598-022-04901-z (PMC8770454; doi:10.1038/s41598-022-04901-z)
Supplement: Supplementary file 1 — Supplementary Information. [file 41598_2022_4901_MOESM1_ESM.docx]

[Supplementary information]

Physically Unclonable Functions Taggant for Universal Steganographic Prints

Takao Fukuoka^1*^, Yasushige Mori^2^, Toshiya Yasunaga^3^, Kyoko Namura^1^, Motofumi Suzuki^1^, and Akinobu Yamaguchi^4*^

^1^ Department of Micro Engineering, Kyoto University, Kyoto Daigaku-Katsura, Nishikyo-ku, Kyoto 615-8540, Japan

^2^ Department of Chemical Engineering and Materials Science, Doshisha University, 1-3 Tatara Miyakodani, Kyotanabe-shi, Kyoto 610-0394, Japan

^3^ Laboratory of Pharmaceutical Engineering, School of Pharmacy, Aichi Gakuin University, 1-100 Kusumoto-cho, Chikusa-ku, Nagoya, Aichi 464-8650, Japan

^4^ Laboratory of Advanced Science and Technology, University of Hyogo, 3-1-2 Kouto, Kamigori, Ako-gun, Hyogo 678-1205, Japan

*Materials & Correspondence : T. F. (tak_f@mpe.me.kyoto-u.ac.jp) & A. Y. (yamaguti@lasti.u-hyogo.ac.jp)

SERS signals of an Amabie Stamp were measured with NanoPhoton RAMANtouch. The Amabie Stamp was printed with nanobeacon ink. The Raman signals were measured by 10 times Object lens with 785 wavelength laser. The laser power was 50 mW and its expose time was 1 sec. We measured three locations with different ink characteristics. These results are shown in SFig. 1 – 3.


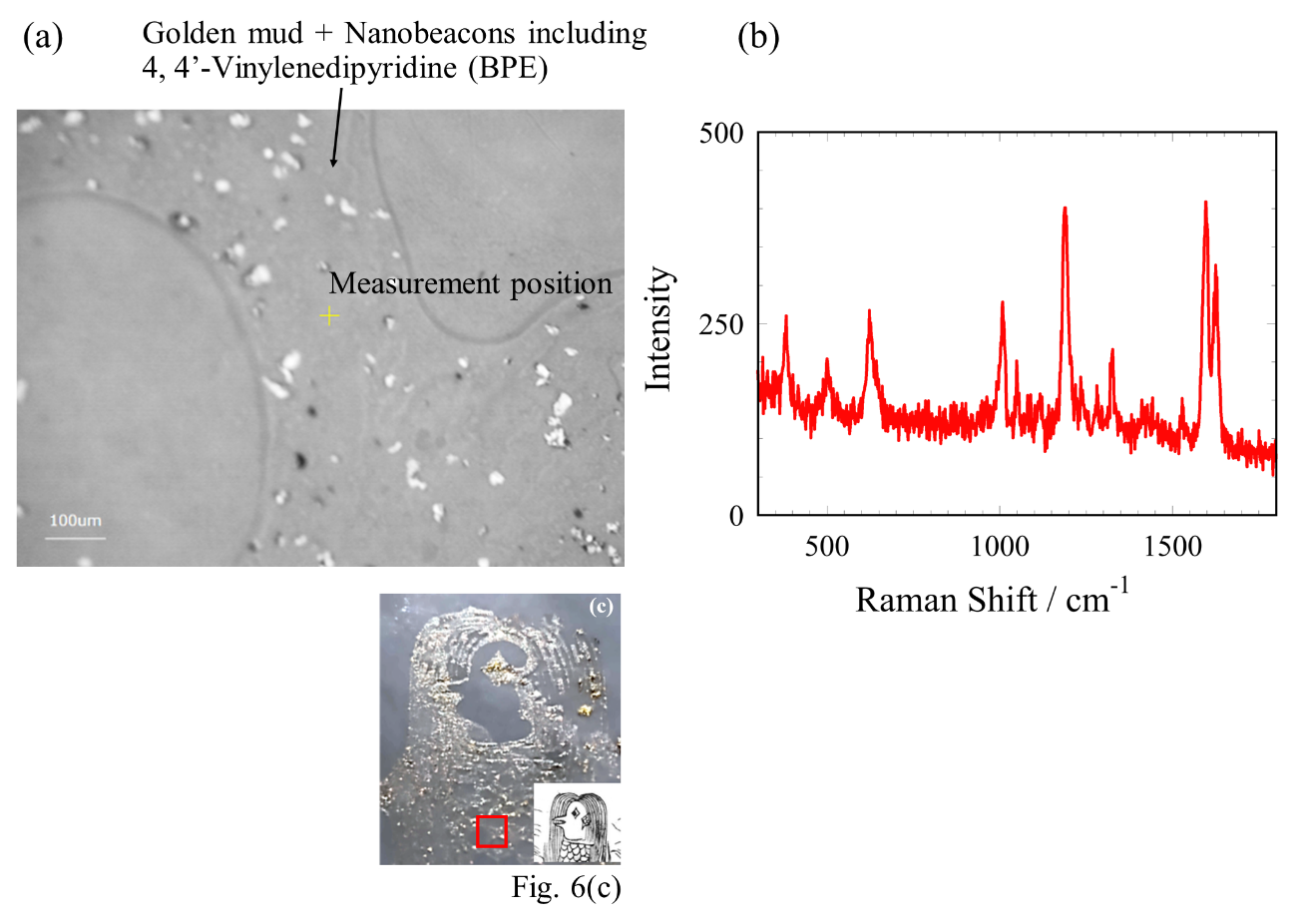


SFig. 1 (a) Optical image of measurement position, corresponding to the magnified image from the area surrounded by red solid line in Fig. 6(c). The Raman measurement position is on the Plain area, corresponding to the yellow cross mark. The position was Varnish. (b) SERS signal can be detected from only the paste.


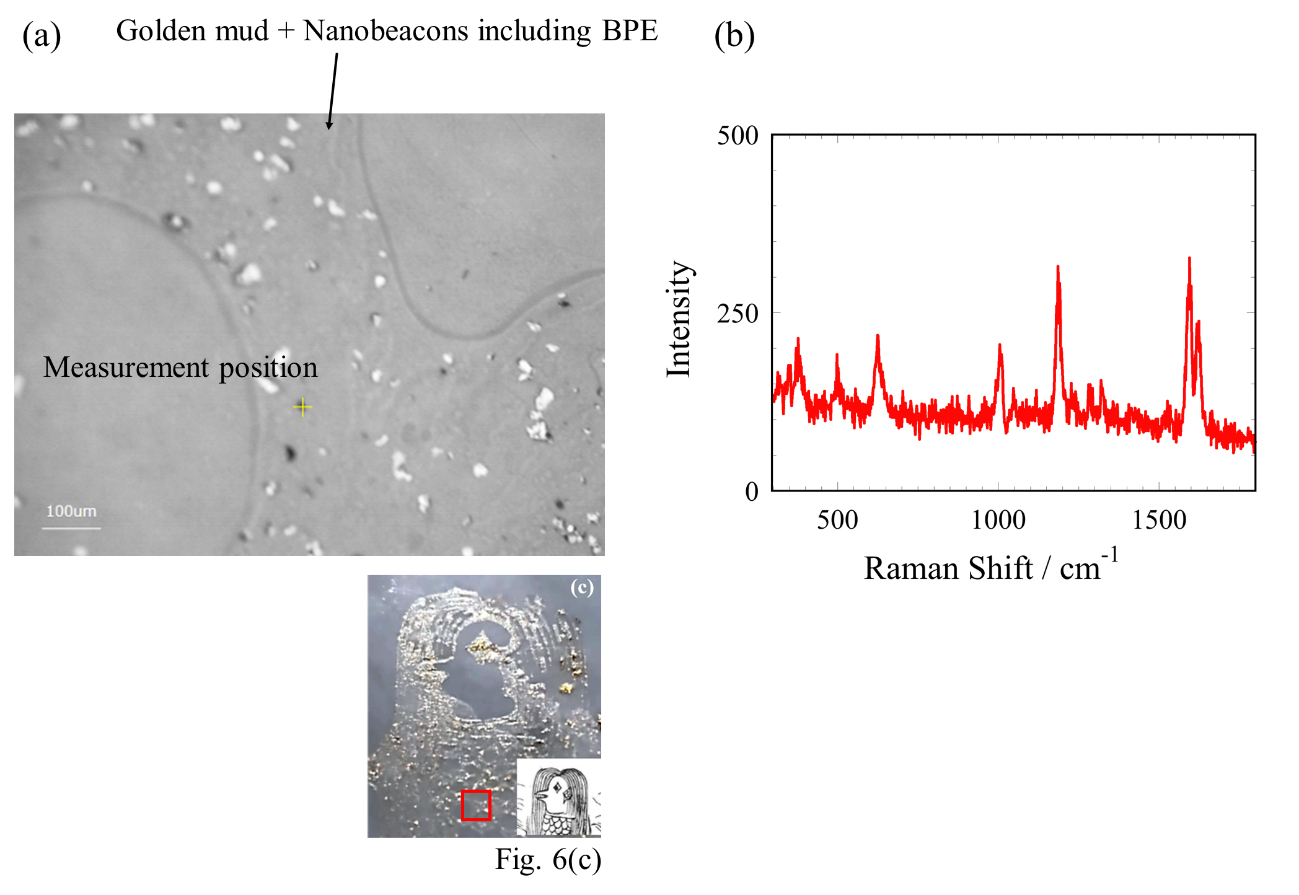


SFig. 2 (a) Optical image of measurement position, corresponding to the magnified image from the area surrounded by red solid line in Fig. 6(c). The Raman measurement position is on the Granules, which was considered to be a part of pigment of Gold mud. The laser spot, that is the yellow cross mark position, is larger than the granule or vanish. (b) SERS signal can be detected from the measurement position.


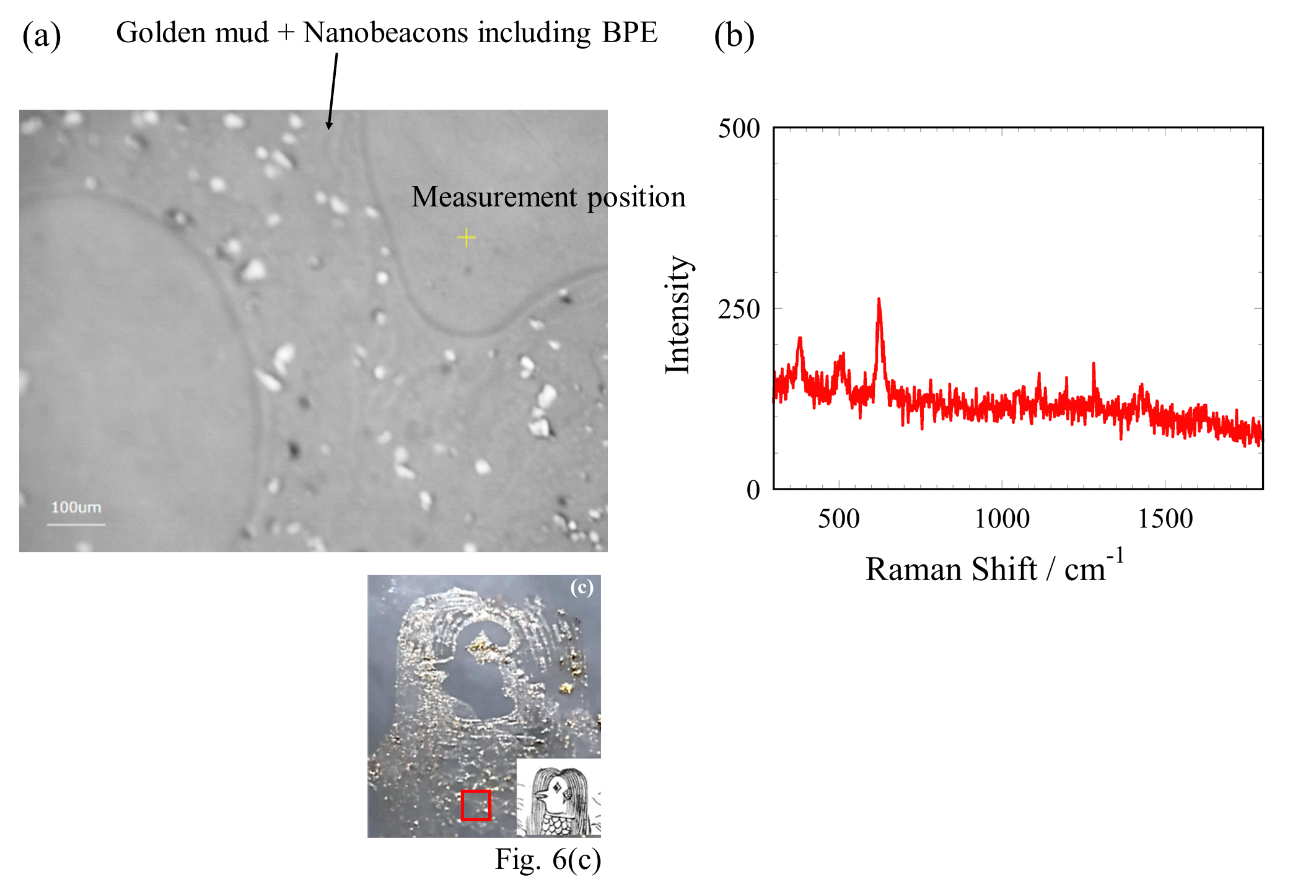


SFig. 3 (a) Optical image of measurement position, corresponding to the magnified image from the area surrounded by red solid line in Fig. 6(c). The Raman measurement position is on background, which was a paper without stamping. (b) The measured Raman signal was considered to be just anatase. No SERS signal can be detected.
